# GATA6 is essential for endoderm formation from human pluripotent stem cells

**DOI:** 10.1242/bio.026120

**Published:** 2017-06-12

**Authors:** J. B. Fisher, K. Pulakanti, S. Rao, S. A. Duncan

**Affiliations:** 1 Department of Cell Biology, Neurobiology and Anatomy, Medical College of Wisconsin, Milwaukee, WI 53226, USA; 2 Blood Center of Wisconsin, Milwaukee, WI 53226, USA; 3 Division of Pediatric Hematology, Oncology, and Blood and Marrow Transplant, Medical College of Wisconsin, Milwaukee, WI 53226, USA; 4 Department of Regenerative Medicine and Cell Biology, Medical University of South Carolina, Charleston, SC 29425, USA

**Keywords:** GATA, Endoderm development, Pluripotent stem cell differentiation, Transcriptional control

## Abstract

Protocols have been established that direct differentiation of human pluripotent stem cells into a variety of cell types, including the endoderm and its derivatives. This model of differentiation has been useful for investigating the molecular mechanisms that guide human developmental processes. Using a directed differentiation protocol combined with shRNA depletion we sought to understand the role of GATA6 in regulating the earliest switch from pluripotency to definitive endoderm. We reveal that GATA6 depletion during endoderm formation results in apoptosis of nascent endoderm cells, concomitant with a loss of endoderm gene expression. We show by chromatin immunoprecipitation followed by DNA sequencing that GATA6 directly binds to several genes encoding transcription factors that are necessary for endoderm differentiation. Our data support the view that GATA6 is a central regulator of the formation of human definitive endoderm from pluripotent stem cells by directly controlling endoderm gene expression.

## Abstract (precis)

**Summary:** Using the differentiation of huESCs as a model for endoderm formation, we reveal that the transcription factor GATA6 regulates the onset of endoderm gene expression and is required for its viability.

## INTRODUCTION

Studies in zebrafish, frogs, nematodes and mice have identified key growth factors and transcriptional regulators that promote endoderm specification and development of endoderm-derived tissues. In this context, researchers have intensely examined the contribution of the GATA binding protein family. GATA binding proteins are transcription factors that contain two highly conserved zinc finger DNA-binding domains that recognize an (A/T)GATA(A/G) consensus nucleotide sequence (Molkentin, 2000). GATA family members are widely expressed and perform diverse biological functions (Oka et al., 2007). GATA4, GATA5 and GATA6 are the most prominently expressed GATA binding proteins during the development of both extra-embryonic and definitive endoderm cell lineages in mouse embryos (Laverriere et al., 1994; Morrisey et al., 1996). GATA4 and GATA6 have been reported as necessary for the development and function of a number of endoderm-derived tissues and cells including hepatocytes (Matsuda et al., 1994; Zhao et al., 2005; Watt et al., 2007), intestinal epithelium (Bossard and Zaret, 1998; Belaguli et al., 2007; Bosse et al., 2007; Battle et al., 2008; Beuling et al., 2011, 2012; Walker et al., 2014), endocrine and exocrine pancreas (Ketola et al., 2004; Carrasco et al., 2012; Xuan et al., 2012; Martinelli et al., 2013), and lung (Keijzer et al., 2001; Yang et al., 2002; Ackerman et al., 2007). During endoderm development in *Xenopus* and zebrafish, GATA4, 5 and 6 appear to act redundantly (Weber et al., 2000; Afouda et al., 2005; Peterkin et al., 2007) to reinforce endoderm fate downstream of Nodal signaling (Alexander and Stainier, 1999; Rodaway et al., 1999; Weber et al., 2000; Reiter et al., 2001). In *C.* *elegans*, mutation of the *end-1* gene results in loss of the endoderm, implying that a requirement for GATA factors in regulating endoderm development is evolutionarily conserved (Zhu et al., 1997). Studies in mice revealed that germline deletion of GATA4 or GATA6 results in early embryonic lethality due to defects in the extra-embryonic endoderm, a cell type that contributes to the yolk sac and is distinct from the definitive endoderm of the fetus (Kuo et al., 1997; Molkentin et al., 1997; Koutsourakis et al., 1999; Morrisey et al., 1998). Providing GATA null embryos with a wild-type extra-embryonic endoderm through tetraploid complementation circumvented the lethality, and revealed roles for GATA4 and GATA6 in heart and liver development (Narita et al., 1997; Zhao et al., 2005, 2008; Watt et al., 2007).

The fact that GATA4 and GATA6 regulate the development of the extra-embryonic endoderm has complicated the study of the molecular mechanisms through which GATA factors contribute to the formation of the definitive endoderm. However, molecular and biochemical analyses, specifically of GATA4, have revealed that the GATA proteins may act as pioneer factors at the earliest stages of definitive endoderm development (Bossard and Zaret, 1998; Cirillo and Zaret, 1999; Zaret, 1999; Cirillo et al., 2002; Zaret et al., 2008). Protocols that recapitulate early stages of mammalian development have been established to promote the differentiation of human pluripotent stem cells to definitive endoderm in culture (D'Amour et al., 2005). The availability of a pluripotent stem cell model that mirrors the development of endoderm in culture offers the potential to help investigators define the molecular mechanisms that promote the formation of endoderm in humans. In this study, we use the differentiation of human pluripotent stem cells to provide evidence that GATA6 acts upstream of GATA4 and is essential for the generation of definitive endoderm by human pluripotent stem cells. GATA6 depletion during definitive endoderm formation results in apoptosis of the differentiating cells concomitant with a loss of endoderm gene expression. GATA6 occupies genomic sequences in a diverse array of genes expressed in the endoderm and is necessary for expression of several transcription factors known to be essential for definitive endoderm development.

## RESULTS

### Onset of GATA4 and GATA6 expression is coincident with the beginning of endoderm gene expression

**Fig. 1.**
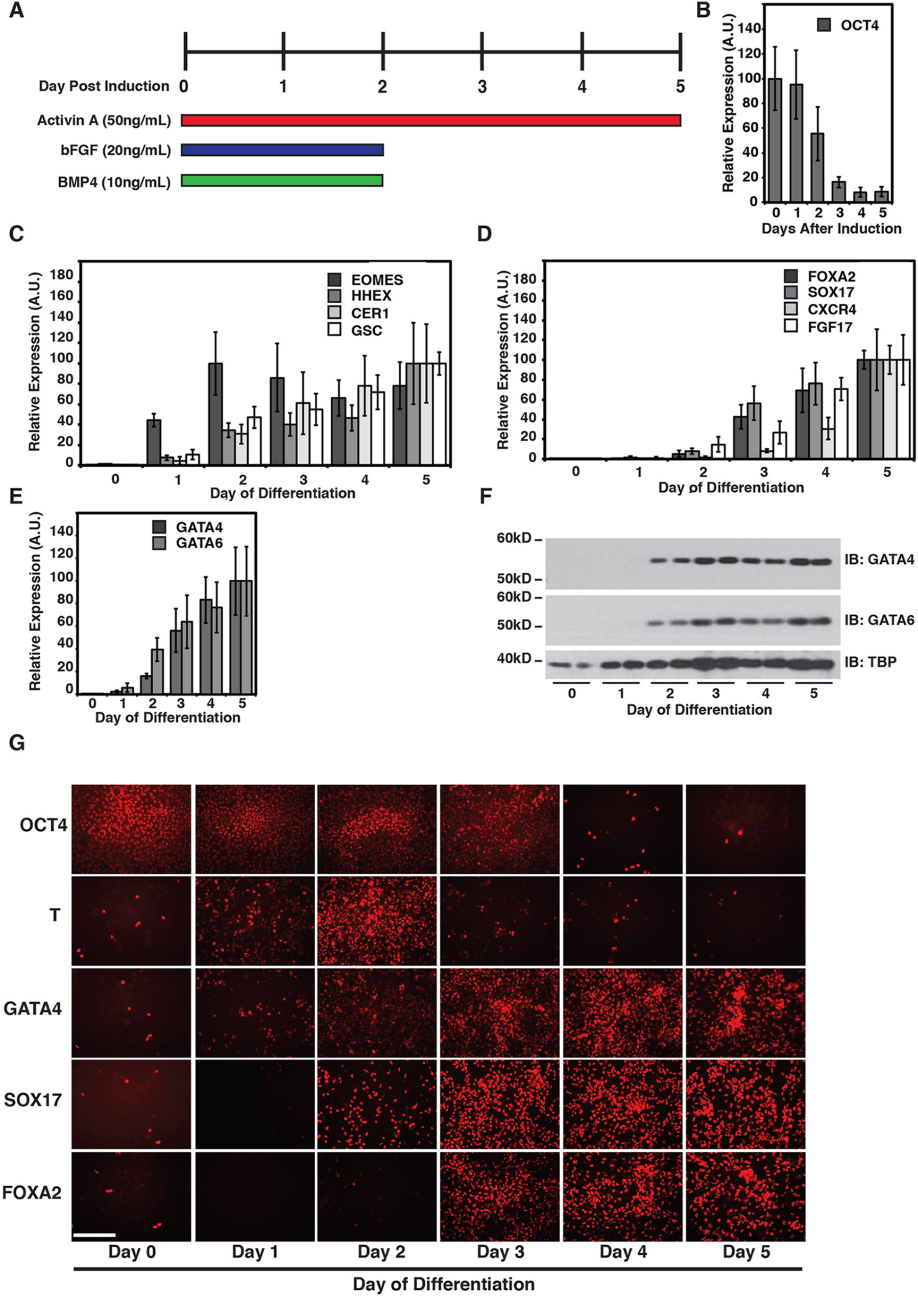
**Directed differentiation of human pluripotent cells recapitulates the early stages of endoderm development.** (A) Schematic of the approach used for differentiating pluripotent cells into definitive endoderm cells. (B-E) Bar graphs showing the results of RT-qPCR analysis of pluripotent H1-huESCs (day 0) or cells through days 1–5 of differentiation. Error bars represent the standard error of the mean for three independent differentiations. Levels of mRNA encoding (B) *OCT4* (pluripotency marker), (C) *EOMES*, *HHEX*, *CER1* and *GSC* (early mesendoderm markers), (D) *FOXA2*, *SOX17*, *CXCR4* and *FGF17* (endoderm markers), and (E) *GATA4* and *GATA6*. (F) Immunoblot analyses of nuclear extracts collected from huESCs (day 0) or differentiating endoderm (days 1–5) reveals the appearance of GATA4 and GATA6 protein during endoderm differentiation. TATA-Binding Protein (TBP) was used as a loading control. (G) Micrographs of immunocytochemistry performed on pluripotent cells (day 0) or differentiating endoderm (days 1–5) using antibodies to detect the pluripotency marker OCT4, the mesendoderm marker Brachyury (T) and definitive endoderm markers GATA4, FOXA2 and SOX17. Images shown are representative of three independent differentiations. Scale bar:100 µm.

Given that GATA4 and GATA6 are transcription factors with well-established roles in the differentiation of a number of cell types that are crucial for organ development and function (Kuo et al., 1997; Molkentin et al., 1997; Morrisey et al., 1998; Watt et al., 2004; Holtzinger and Evans, 2005; Zhao et al., 2005, 2008; Decker et al., 2006; Sodhi et al., 2006; Kanematsu et al., 2007; Holtzinger et al., 2010; van Berlo et al., 2010; Beuling et al., 2011; Carrasco et al., 2012; Martinelli et al., 2013; Delgado et al., 2014; Walker et al., 2014), we sought to define the role of these factors in regulating the earliest formation of the definitive endoderm in human cells. We previously reported a protocol for the directed differentiation of pluripotent stem cells into hepatocyte-like cells in which markers of definitive endoderm were expressed 5 days after the onset of differentiation (Fig. 1A) (Si-Tayeb et al., 2010; Mallanna and Duncan, 2013). We first attempted to define the window of the onset of definitive endoderm gene expression during differentiation using this protocol. We measured steady-state levels of mRNAs encoding diagnostic differentiation markers by real-time quantitative polymerase chain reaction (RT-qPCR) in samples collected from pluripotent H1 human embryonic stem cells (huESCs) (day 0) or differentiating endoderm at each day after induction (day 1–5). As anticipated, expression of the pluripotent marker OCT4 steadily decreased as the cells adopted a definitive endoderm identity (Fig. 1B). Expression of the earliest markers of endoderm and mesendoderm, including Eomesodermin (EOMES), Goosecoid Homeobox (GSC), Hematopoietically Expressed Homeobox (HHEX) and Cerberus 1, DAN Family BMP Antagonist (CER1), began within 24 h of induction. Except for EOMES, the mRNA levels of which stayed relatively constant, mRNAs encoding the other markers continued to increase daily (Fig. 1C). Expression of Forkhead Box A2 (FOXA2), SRY-Box 17 (SOX17), C-X-C Motif Chemokine Receptor 4 (CXCR4), and Fibroblast Growth Factor 17 (FGF17), began 48–72 h after induction, and also continued to increase throughout the differentiation period (Fig. 1D). GATA4 and GATA6 mRNA levels could first be reliably detected by day 2 after induction and continued to rise throughout the differentiation period, coincident with the accumulation of other endoderm markers (Fig. 1E).

Similar to mRNA distribution, immunoblotting revealed the presence of both GATA4 and GATA6 protein 2 days after induction (Fig. 1F). Immunostaining was then performed to determine the distribution of cells at each day after induction that were positive for the pluripotency marker OCT4, the mesendoderm marker brachyury (T), and the endoderm markers SOX17, FOXA2 and GATA4 (Fig. 1G). As expected, the numbers of OCT4-positive cells were high in the pluripotent samples, but steadily declined throughout the differentiation time course, and at days 4 and 5 only a few positive cells remained. Consistent with RT-qPCR and immunoblotting data, the number of SOX17- and GATA4-positive cells was low to undetectable prior to induction, followed by a marked increase at day 2 that continued to rise throughout differentiation. We also observed an increase in the number of cells expressing the known endoderm factor FOXA2 beginning at days 3–4 after induction (D'Amour et al., 2005). In contrast to the distribution of endoderm proteins, the percentage of cells that express brachyury (T), which reinforces the mesodermal lineage, is high within 24 h, but rapidly declines after day 2 of differentiation. These data demonstrate that the directed differentiation of human pluripotent cells to definitive endoderm recapitulates the events observed from animal studies and supports previous findings by others (Yasunaga et al., 2005; McLean et al., 2007). Importantly, these analyses confirm that GATA4 and GATA6 are expressed at the onset of definitive endoderm formation using this stem cell model which is consistent with a potential role in converting pluripotent cells to an endoderm fate.

### Depletion of GATA6 causes a loss of endoderm viability

**Fig. 2.**
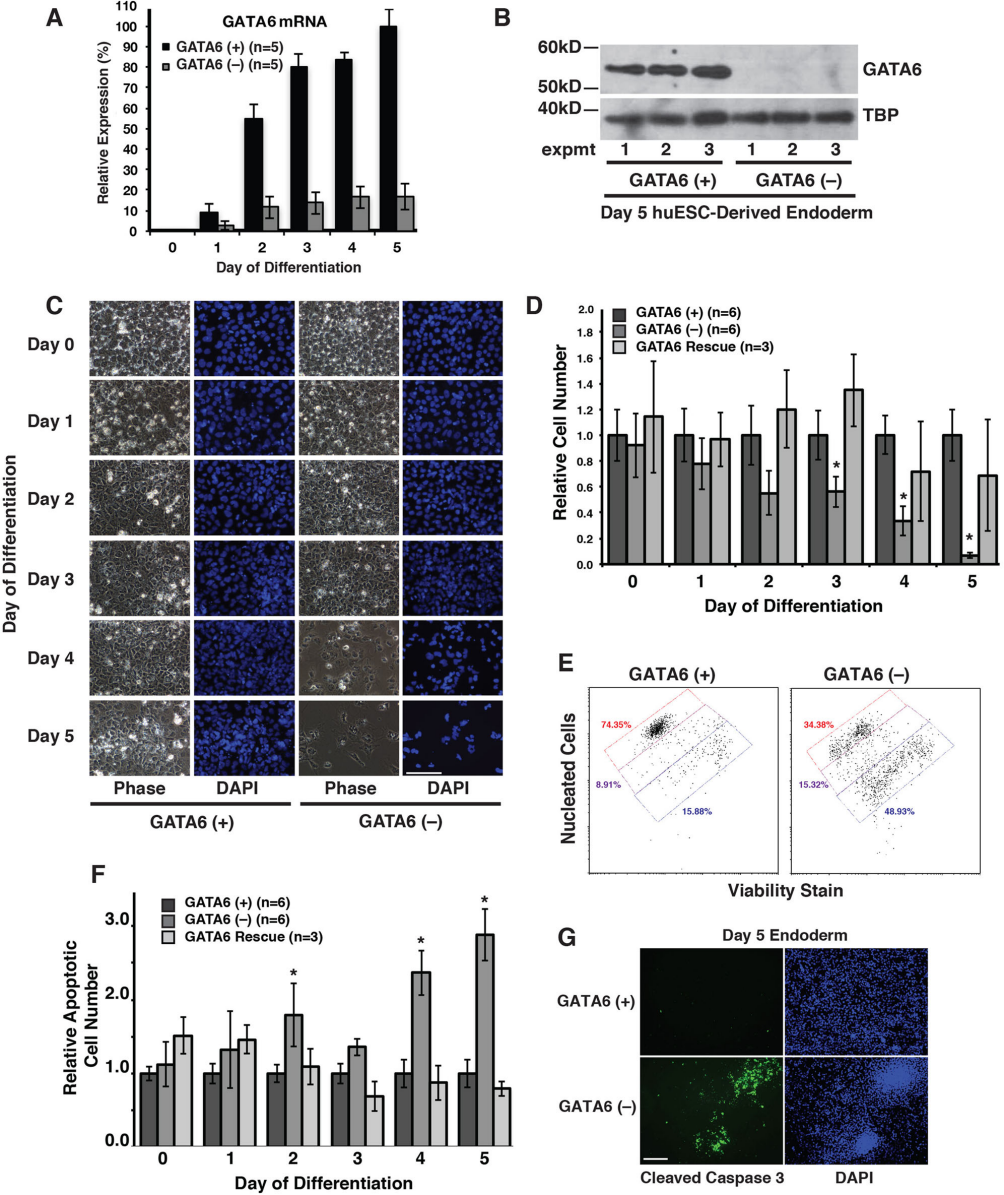
***GATA6* depletion induces apoptosis of definitive endoderm.** (A) Bar graph showing the results of RT-qPCR analysis of control ESCs [GATA6 (+)] and ESCs expressing a *GATA6* shRNA [GATA6 (−)] collected daily during endoderm differentiation (days 0–5). Error bars represent standard error of the mean for five independent differentiations. (B) Immunoblot analysis performed on nuclear extracts from control and *GATA6*-depleted cells harvested at day 5 of endoderm differentiation (*n*=3 independent differentiations). TBP was used as a loading control. (C) Micrographs showing phase-contrast imaging and DAPI staining of GATA6 (+) and GATA6 (−) pluripotent (day 0) or differentiating endoderm cells (days 1–5). Images shown are representative of three independent differentiations. Scale bar: 100 μm. (D,F) Bar graphs showing the results of flow cytometry to identify total cell number (D) and apoptotic cell number (F) during the differentiation of GATA6 (+) (*n*=6 independent differentiations), GATA6 (−) (*n*=6 independent differentiations), and *GATA6–shRNA^indFlagGATA6^* (GATA6 Rescue; *n*=3 independent differentiations) cells. Error bars represent standard error of the mean and significance was determined using Student's *t*-test; *P<0.05. (E) Representative flow cytometry dot plot for one group of day 4 samples showing gating conditions for live nucleated cells (viability dye excluded, red gate), apoptotic (viability dye low, purple gate), and dead enucleated cells (viability dye high, blue gate). (G) Micrographs showing immunostaining to detect the level of the activated Caspase-3 in GATA6 (+) and GATA6 (−) cells at day 4 of differentiation. Images are representative of two independent differentiations. Scale bar: 100 µm.

Analyses of induced pluripotent stem cells (iPSCs) in which GATA4 was depleted by shRNA revealed that GATA4 was dispensable for differentiation of the definitive endoderm from huESCs (Fig. S1). We next pursued the role of GATA6 during definitive endoderm development using a previously published shRNA that was reported to efficiently deplete *GATA6* mRNA in primary bladder smooth muscle cells (Kanematsu et al., 2007). We generated a polyclonal line of huESCs that expressed the *GATA6* shRNA (Fig. 2A). Examination of *GATA6* steady-state mRNA levels by RT-qPCR at days 0 and 5 of differentiation revealed a reduction of >80% of *GATA6* mRNA compared to control cells that we transduced with empty vector (Fig. 2A). The impact of the shRNA was immediate, since even at day 2 of differentiation when GATA6 expression is robust in control cells, the level of *GATA6* mRNA in cells expressing the shRNA was barely above background (Fig. 2A). As expected and in contrast to control cells, immunoblot analyses confirmed that GATA6 protein was undetectable in extracts of *GATA6* shRNA-expressing cells at day 5 of differentiation (Fig. 2B). These data confirm the efficacy of the GATA6 shRNA described by Kanematsu et al. (2007).

During the differentiation of the GATA6-depleted ESCs, we observed a substantial reduction in cell number by days 4 and 5 of differentiation compared to control cells (Fig. 2C). To quantify these changes in cell density, we monitored cell numbers over the course of the differentiation by flow cytometry using a commercial assay (ViaCount) that distinguishes between live, apoptotic and dead cells. We differentiated control (empty vector) and *GATA6* shRNA-treated cells into definitive endoderm, and cells were collected daily over the entire time course. We observed a significant reduction in cell numbers starting at day 3 of differentiation, and by day 5 the population had dropped by around 90% (Fig. 2D). To explain this reduction in cell number, we first compared the rate of cell proliferation during the differentiation of control and GATA6-depleted cells (Fig. S2). The number of proliferating cells was determined using 5-ethynyl-2′-deoxyuridine (EdU) incorporation (Fig. S2A,B) and mitotic cell numbers determined by phospho-histone H3 expression (Fig. S2C). Despite the reduction in cell number, depletion of GATA6 had no effect on the percentage of proliferating cells. We next monitored the accumulation of apoptotic cells throughout endoderm differentiation by flow cytometry again using ViaCount reagent. Representative flow cytometry dot plots reveal the respective live and dead cell populations, while low viability dye cells fall into the apoptosis gate (Fig. 2E). We observed an increase in apoptosis beginning at day 2 after induction (Fig. 2F), coinciding with the onset of GATA6 expression (Fig. 1E,F). The level of apoptosis subsided at day 3 then increased dramatically at days 4 and 5 of differentiation (Fig. 2F). To confirm that the death of cells was due to apoptosis, we performed immunostaining for activated/cleaved caspase-3. At day 4 of differentiation, a substantial number of activated caspase-3-positive cells were present in the *GATA6* shRNA differentiations with very few positive cells found in the control differentiations (Fig. 2G).

We felt it was important to confirm that the observed loss of viability of the endoderm was indeed a consequence of GATA6 depletion and not due to off-target effects of the shRNA. We, therefore, generated a cell line in which a shRNA-resistant *GATA6* cDNA was expressed in H1 ESCs containing the *GATA6* shRNA (*GATA6–shRNA^indFlagGATA6^*, GATA6 Rescue). Expression of the GATA6 shRNA-resistant transgene was controlled through the use of a doxycycline-inducible promoter and inclusion of a FLAG-epitope tag allowed us to distinguish expression of the transgene from endogenous GATA6 (Fig. S3A). We treated the cells with 100 ng/ml doxycycline during days 1–5 of endoderm differentiation and confirmed exogenous GATA6 expression by anti-FLAG immunostaining at day 5 of differentiation (Fig. S3B). A dose response curve revealed that expression of the *GATA6* cDNA was robustly induced in the endoderm (day 5) using 40 ng/ml doxycycline and so we used this concentration in all subsequent experiments (Fig. S3C). Importantly, in the absence of doxycycline, *GATA6* mRNA was below the limit of detection in the *GATA6–shRNA^indFlagGATA6^* endoderm, whereas inclusion of doxycycline resulted in a fivefold overexpression of *GATA6* (Fig. S3D). As shown in Fig. 2D, treatment of the *GATA6–shRNA^indFlagGATA6^* endoderm with doxycycline to induce GATA6 expression abrogated the reduction in cell number observed when GATA6 was depleted. Additionally, in the presence of doxycycline, the number of apoptotic cells associated with GATA6 depletion reverted to those found in the endoderm derived from control cells (Fig. 2F). From these data, we conclude that depletion of GATA6 induces apoptotic cell death of the newly specified definitive endoderm.

### GATA6 is essential for the legitimate expression of endodermal mRNAs

Since GATA factors are transcription factors, we questioned whether depletion of GATA6 during human definitive endoderm formation affects gene expression. We performed oligonucleotide array analyses on control and GATA6-depleted cells at each day of differentiation. We collected data from two independent differentiations (biological replicates, *n*=2) for each time point. First, we defined a set of genes for which expression was induced in wild-type huESC-derived definitive endoderm by establishing mRNA profiles between pluripotent cells and day 5 definitive endoderm cells. Expression levels for each probe set were used to calculate z-scores and ANOVA was performed using Partek software. Levels of mRNAs that were increased with high confidence (z-score ≥3 and *P*≤0.01) in day 5 endoderm compared to pluripotent cells were considered enriched. We focused on genes for which expression increased because we were most interested in identifying positive markers of the endoderm rather than those indicating a loss of pluripotency. Fold changes correlating to z-score and *P*-value cutoffs for each comparison are summarized in Table S3B. We identified 464 unique genes with high confidence for which expression increased in definitive endoderm cells compared to pluripotent stem cells (Table S4A). The list of huESC-derived definitive endoderm-enriched mRNAs comprised of transcription factors (50), cytokines and growth factors (25), cell differentiation markers (20) and protein kinases (19) (Table S4B). Additionally, many of these mRNAs have been previously shown to be expressed in the definitive endoderm of mouse embryos including *Eomes* (Arnold et al., 2008; Costello et al., 2011), *Sox17* (Kanai-Azuma et al., 2002; Choi et al., 2012; Viotti et al., 2014), *Gsc* (Belo et al., 1997), *Hhex* (Martinez Barbera et al., 2000; Bort et al., 2006; Rankin et al., 2011), *Cer1* (Bouwmeester et al., 1996; Wang et al., 2012) and *Gata4* (Rojas et al., 2010), and have been used as markers of definitive endoderm during directed differentiation protocols (D'Amour et al., 2005; Yasunaga et al., 2005; McLean et al., 2007; Teo et al., 2011).

**Fig. 3.**
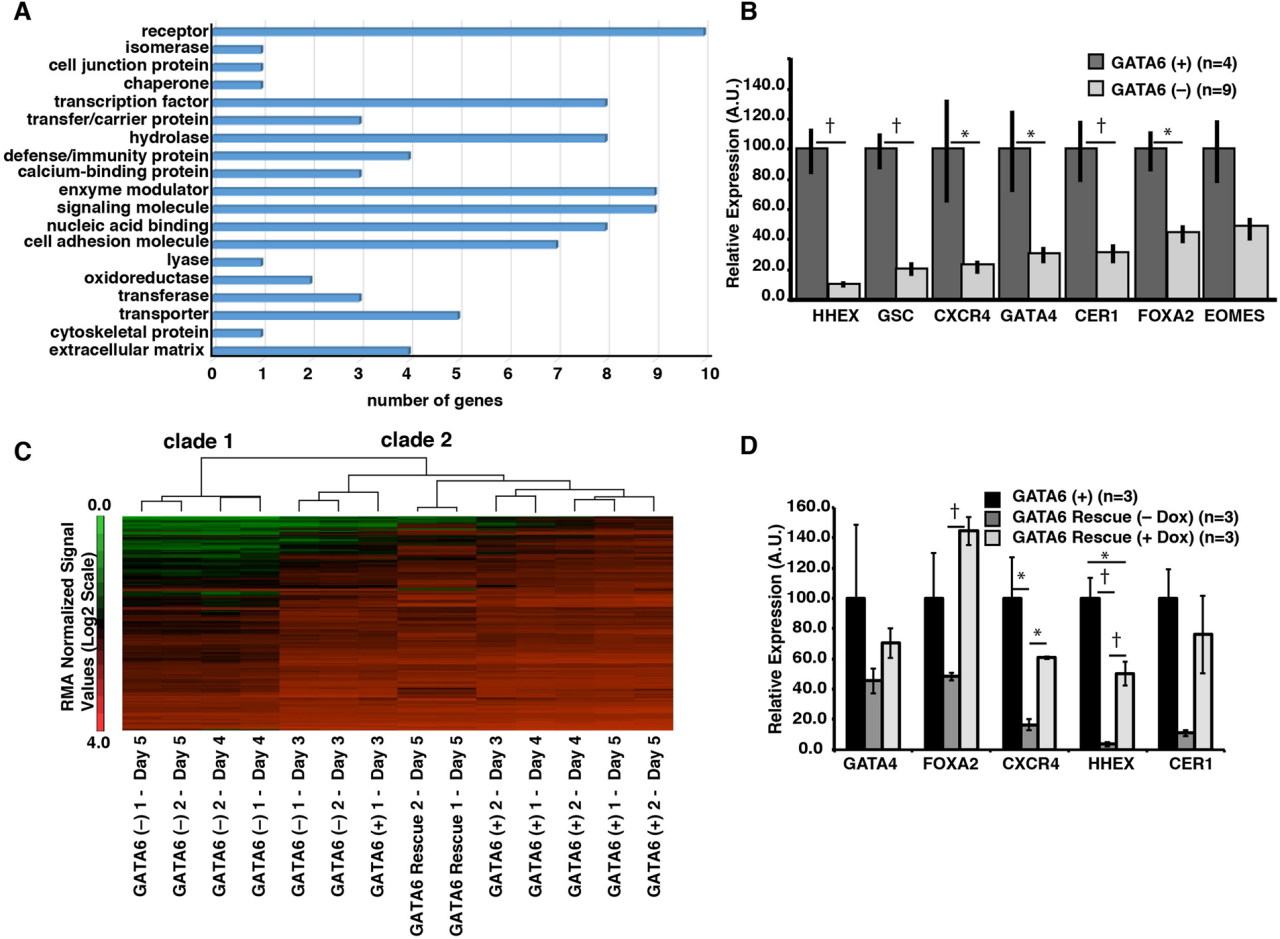
***GATA6* depletion causes loss of definitive endoderm gene expression.** (A) Bar graph showing results of gene ontology analysis using PANTHER of genes for which expression was affected by GATA6 depletion at day 3 of differentiation. (B) RT-qPCR analysis of GATA6 (+) and GATA6 (−) cells collected 5 days after induction. (C) Hierarchical cluster analysis of the mRNA levels of 464 genes contained within the definitive endoderm-enriched gene list in GATA6 (+) and GATA6 (−) cells at day 3, day 4 and day 5 of differentiation, as well as in *GATA6–shRNA^indFlagGATA6^* (GATA6 Rescue+Dox) cells at day 5 of differentiation. (D) RT-qPCR analysis of control and *GATA6* shRNA rescue line with or without doxycycline. Error bars represent the standard error of the mean for the number of independent differentiations indicated. Statistical significance was determined via Student's *t*-test; *P<0.05, †*P*<0.01.

To define the impact of GATA6 depletion on endoderm gene expression, we differentiated control and GATA6-depleted cells to days 3, 4, and 5 and measured changes in mRNAs encoding huESC-derived definitive endoderm-enriched proteins by oligonucleotide array analyses. For these studies, we examined all mRNAs from the definitive endoderm-enriched gene list that exhibited a z-score <−3 or >+3 and had a *P*-value≤0.01 in the GATA6-depleted cells compared to control cells. Again, we performed all analyses on two independent differentiations at each time point (biological replicates, *n*=2). At day 3 of differentiation only a modest number (14.4%) of definitive endoderm-enriched mRNAs were affected by GATA6 depletion, with 11 (2.4%) exhibiting increased levels and 56 (12.1%) exhibiting decreased levels (Table S4C). Despite the fact that relatively few genes were affected at day 3 of differentiation, gene ontology analyses revealed that they encoded a range of functions commonly associated with cell differentiation and viability, such as receptors, enzymes, DNA binding proteins and transporters. Moreover, several of the affected genes encoded transcription factors (including GATA6, SRY, FOXQ1, IFI16, ZFPM2, BHLHE22, TAF9B and MNX1) and signaling molecules (CD48, NCR1, MCF2L2, TNC, UPK1B, SEMA3D, S100A14, ODZ2 and S100A16) (Fig. 3A; Table S4B). The impact of GATA6 depletion became more striking at day 4 of differentiation when 248 (53.4%) definitive endoderm-enriched mRNAs were affected, and of those, 247 mRNAs (53.2%) decreased while only one mRNA (0.2%) increased (Table S4D). Similarly, at day 5 of differentiation, the level of 297 (64%) mRNAs were altered, with 295 (63.6%) diminished and only 2 (0.4%) elevated (Table S4E). Finally, we confirmed the accuracy of the array analyses by performing RT-qPCR on control and GATA6-depleted cells at day 5 of differentiation to detect mRNAs encoding proteins that are typically used to characterize endoderm (HHEX, GSC, CXCR4, GATA4, CER1, FOXA2, EOMES) and have important roles in controlling endoderm fate (Fig. 3B). All of these mRNAs were dramatically reduced when GATA6 was depleted. Such a broad impact on the expression of endodermal genes is consistent with loss of the endoderm lineage during the differentiation of GATA6-depleted huESCs at day 5 of differentiation.

To ensure that the observed changes in gene expression were a consequence of loss of GATA6, we asked whether the introduction of the GATA6 shRNA-resistant transgene (*GATA6–shRNA^indFlagGATA6^*) could revert the expression profile to more closely match that of control cells. We induced expression of the GATA6 cDNA in GATA6–depleted cells during days 1–5 of differentiation and determined expression profiles by oligonucleotide array analyses at day 5. Unsupervised hierarchical cluster analyses were performed using the endoderm gene set from days 3, 4 and 5 control and GATA6-depleted cells, and day 5 *GATA6–shRNA^indFlagGATA6^* cells (+doxycycline days 1–5). The entire dataset formed two major clades (clade 1 and clade 2) (Fig. 3C). As expected, all of the control samples clustered, consistent with the cells adopting an endoderm fate (clade 2) (Fig. 3C). At day 3, the GATA6-depleted cells also segregated into clade 2, which is consistent with the observation that expression of only a few mRNAs was affected by the loss of GATA6 at this stage of differentiation. However, at days 4 and 5, the GATA6–depleted samples formed an independent clade (clade 1) (Fig. 3C), confirming that the loss of GATA6 has a dramatic impact on the fate of the endoderm. In contrast to the GATA6-depleted cells, when we examined day 5 samples from *GATA6–shRNA^indFlagGATA6^* cells treated with doxycycline, the expression profile clustered in clade 2 along with the control cells (Fig. 3C; Table S4G) indicating that expression of GATA6 cDNA was sufficient to restore endoderm differentiation. Of the 297 definitive endoderm-enriched mRNAs for which expression was altered by GATA6 depletion, 213 (72%) were no longer significantly affected (Table S4F) when exogenous GATA6 was expressed. Expression of *APLNR* and *DKK1*, the only definitive endoderm-enriched genes that were elevated by GATA6-depletion, were also reduced close to control levels. Importantly, the level of mRNAs encoding key definitive endoderm transcription factors, such as *FOXA2*, *GATA4*, *SOX17*, *HHEX* and *GSC*, were no longer significantly changed from control cells. RT-qPCR was used to validate that the level of a subset of definitive endoderm-enriched mRNAs was rescued by exogenous expression of the shRNA-resistant *GATA6* cDNA (Fig. 3D). From these data, we conclude that GATA6 is required for expression of characteristic endoderm markers.

### GATA6 occupies binding sites within genes encoding transcription factors that regulate definitive endoderm fate

**Fig. 4.**
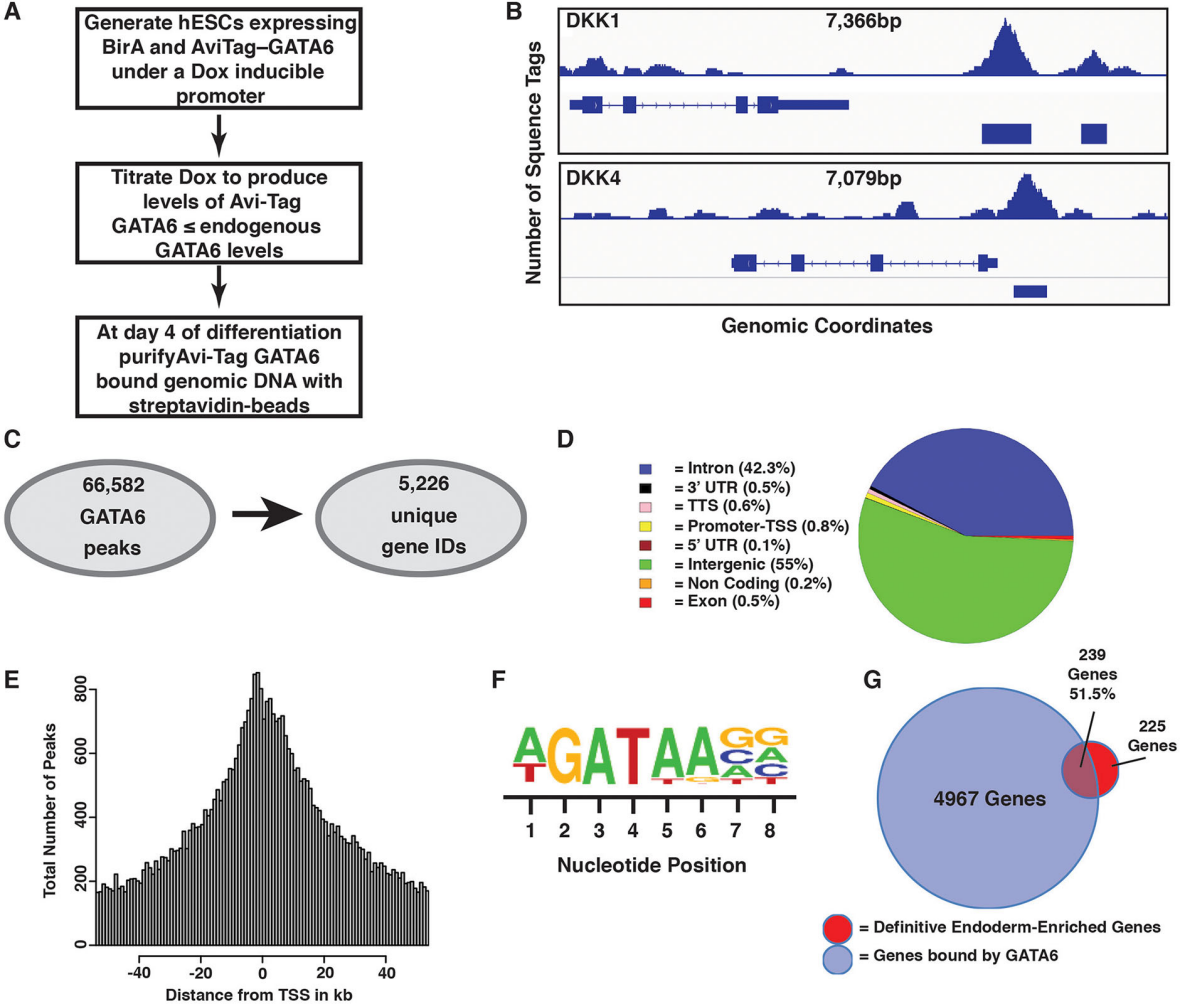
**GATA6 is bound to definitive endoderm-enriched genes.** (A) Workflow used to identify GATA6 occupied sequences throughout the genome at day 4 of differentiation. (B) Two examples showing the position of GATA6-enriched sequences (peaks) within the *DKK1* and *DKK4* genes. (C) Synopsis of the number of GATA6 peaks identified by ChIP-seq and the corresponding number of unique genes. (D) Pie chart showing the percentages of GATA6 binding across annotated genomic regions. (E) Distribution of GATA6 peaks relative to the transcriptional start site of the nearest gene. (F) Consensus GATA6 binding site across all peaks. (G) Venn diagram showing the overlap between genes bound by GATA6 and definitive endoderm-enriched genes.

Despite the observation that changes in gene expression in the GATA6-depleted cells coincided with the onset of GATA6 expression in control endoderm, it remained unclear whether any of the affected genes were direct targets of GATA6. We, therefore, performed chromatin immunoprecipitation followed by DNA sequencing (ChIP-seq) on endoderm derived from control hESCs at day 4 of differentiation. Despite extensive testing, we were unable to identify antibodies that specifically precipitated GATA6 and did not recognize the highly homologous GATA4 and GATA5 proteins. To circumvent the lack of suitable antibodies, we used an epitope-tagged version of GATA6 following the approach presented in Fig. 4A. We tagged human GATA6 with a 15 amino acid sequence (AviTag) recognized by *E. coli* biotin ligase (BirA) (Fig. S4A) (Kim et al., 2009; Wang et al., 2009; Rao et al., 2010). The expression of BirA is necessary and sufficient to biotinylate the AviTag epitope, so AviTag–GATA6–chromatin complexes can be precipitated using streptavidin. We generated a huESC line that expressed AviTag–hGATA6 along with BirA. The AviTag–hGATA6 and BirA proteins were encoded by a T2A polycistronic RNA and were expressed from a doxycycline-inducible promoter (Fig. S4A). We also generated a control huESC line that only expressed BirA. The doxycycline concentration was optimized to ensure that the AviTag–GATA6 transgene was expressed at a level that was no greater than the endogenous GATA6 to reduce the possibility that the AviTag–hGATA6 would bind to ectopic sites in the genome. Of the total GATA6 present in endoderm derived from AviTag–hGATA6 huESCs, 25% was AviTag–GATA6 and 75% was endogenous GATA6 (Fig. S4B-E).

Being satisfied that the AviTag–GATA6 levels were within a physiologically relevant range, we proceeded to precipitate chromatin from definitive endoderm derived from the BirA only control and the GATA6–BirA cells and subject it to high throughput sequencing. We focused on day 4 of differentiation because this was when we first observed a significant impact of GATA6 depletion on endoderm gene expression. As expected, streptavidin precipitation of endoderm from control huESCs that expressed BirA alone failed to yield enough DNA for analysis. This result confirmed the specificity of the BirA for the AviTag sequence. In contrast to the control cells, we identified 66,582 peaks when we precipitated chromatin from endoderm derived from huESCs containing both AviTag–hGATA6 and BirA (Table S5A). Fig. 4B shows an example of enrichment at the DKK1 and DKK4 genes that have roles in regulating WNT signaling. These GATA6-occupied sequences mapped to 7104 genes (within 10 kB of the transcription start site) corresponding to 5226 unique genes (Fig. 4C; Table S5B,C). GATA6 predominantly bound sequences within intergenic and intronic regions with a relatively small percentage bound to proximal promoters (−1 kb to +100 bp of the transcription start site) (Fig. 4D), which is consistent with a previously published report (Tsankov et al., 2015). We confirmed these data by defining the average distance of the position of GATA6 bound sites compared to the gene's transcriptional start site (Fig. 4E). The consensus binding site of all precipitated sequences, identified using HOMER software, revealed that it contained a canonical GATA factor recognition sequence, (A/T)GATA(A), which gave us confidence in the fidelity of the experimental design (Fig. 4F). Our expression profiling indicated that GATA6 depletion caused a large-scale reduction in expression of definitive endoderm mRNAs. Combined with the extensive genomic occupancy exhibited by GATA6, we reasoned that GATA6 was directly regulating the expression of genes expressed in the endoderm. As anticipated, of the 464 genes for which expression was enriched in definitive endoderm, 239 (51.5%) exhibited GATA6 occupancy (Fig. 4G; Table S5D).

**Fig. 5.**
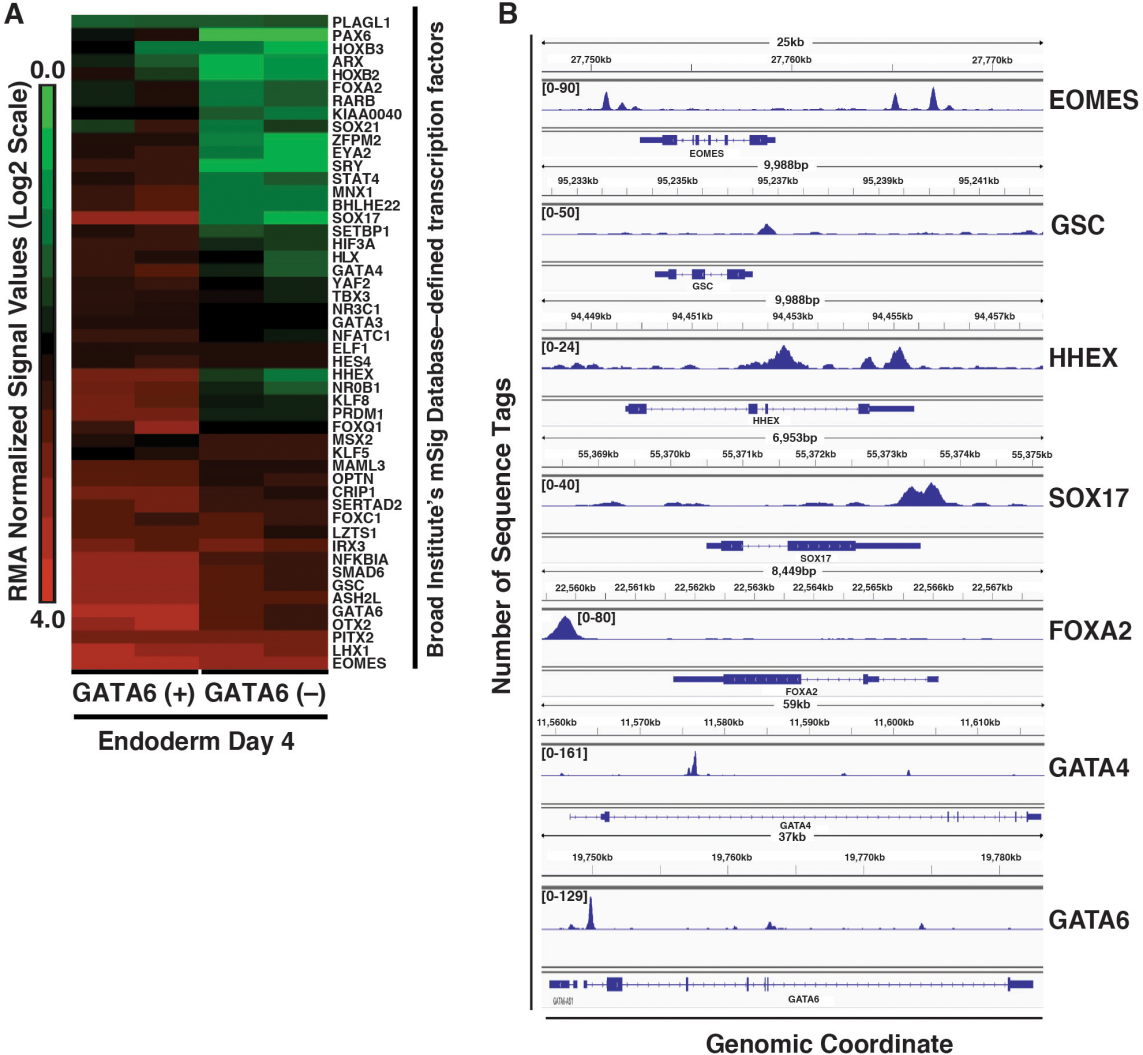
**GATA6 directly binds genes encoding members of the endoderm transcription factor network.** (A) Heatmap showing relative mRNA levels encoding definitive endoderm-enriched transcription factors within GATA6 (+) and GATA6 (−) cells at day 4 of endoderm differentiation. The huESC-derived definitive endoderm-enriched genes shown were identified as transcription factors by the Broad Institute's MSig Database (see Table S4B,H). (B) Integrated genome viewer captures showing representative definitive endoderm-enriched transcription factors that exhibit GATA6 occupancy.

We had demonstrated in Fig. 3 that depletion of GATA6 reduced the level of mRNAs encoding EOMES, GSC, HHEX, FOXA2 and GATA4, all of which have important roles in controlling the formation and differentiation of definitive endoderm (Martinez Barbera et al., 2000; Bjornson et al., 2005; Bort et al., 2006; Zhao et al., 2008; Costello et al., 2011; Rankin et al., 2011). We, therefore, determined the extent to which depletion of GATA6 disrupted endoderm transcription factor expression by analyzing our transcriptome data using a Molecular Signatures Database (MSig, http://software.broadinstitute.org/gsea/msigdb/). Of the genes that had enriched expression in huESC-derived definitive endoderm compared to pluripotent cells, we found that 50 encoded transcription factors (Table S4B). We next examined the mRNA levels encoding these factors in control and GATA6-depleted day 4 endoderm to determine whether GATA6 was necessary for their expression. We chose to examine day 4 endoderm because although there is an increase in apoptotic cell number at this stage, there still exists a substantial number of endodermal cells (Fig. 2). As anticipated, of the 50 definitive endoderm-enriched transcription factors, 46 exhibited some degree of reduction in mRNA levels when we depleted GATA6 (Fig. 5A; Table S4H). Of note, in addition to EOMES, GSC, HHEX, FOXA2 and GATA4, expression of SOX17, which plays a central role in regulating endoderm fate and morphogenesis (Kanai-Azuma et al., 2002; Sinner et al., 2004; Spence et al., 2009; Choi et al., 2012; Viotti et al., 2014), was also dependent on GATA6. Finally, we addressed whether any of the genes encoding these definitive endoderm-enriched transcription factors were occupied by GATA6 by examining our ChIP-seq data. Of the 50 transcription factor genes studied, 25 (50%) contained GATA6 bound sequences (Table S5E). These included EOMES, GSC, HHEX, SOX17, FOXA2 and GATA4 (Fig. 5B). To confirm our results, we compared the peaks that we observed in our ChIP-seq study with those of a previously published study (Tsankov et al., 2015). We observed substantial overlap between the GATA6 bound regions in both studies (Fig. S5A), although our study identified a greater number of GATA6-occupied sequences. Additionally, we observed overlapping peaks at key endoderm transcription factors (Fig. S5B). Based on these data, we conclude that GATA6 directly regulates expression of several transcription factors that establish and maintain endoderm fate.

## DISCUSSION

Cumulatively, our results demonstrate that GATA6 acts upstream of GATA4 to regulate expression of genes that ensure the viability of the definitive endoderm. We characterized endodermal gene expression during directed differentiation of huESCs, and demonstrated that depletion of GATA6 causes apoptosis of the nascent endoderm and a reduction in definitive endoderm gene expression. Consistent with the loss of gene expression, we revealed that GATA6 occupies the presumptive transcriptional regulatory regions of many definitive endoderm-enriched genes. Genes bound by GATA6 included several that encode transcription factors with known roles in endoderm development. One caveat that must be recognized is that the reduction in gene expression could be explained in part by the loss of endoderm cells at late stages of differentiation. However, at day 3 of differentiation, despite the observation that expression of several transcription factors is affected by GATA6 depletion, the impact on cell viability is modest (Fig. 2). Combined with the fact that many endoderm-expressed genes are occupied by GATA6 we favor the view that GATA6 directly regulates expression of endoderm transcription factors.

Recently two groups reported the use of GATA6 deficient human pluripotent stem cells to study the role of GATA factors during pancreatic development (Shi et al., 2017; Tiyaboonchai et al., 2017). Tiyaboonchai et al. found that loss of GATA6 caused an increase apoptosis of nascent endoderm (Tiyaboonchai et al., 2017). Although not the focus of the study, the authors demonstrated that the loss of endoderm could be rescued by expression of GATA6, other GATA family members GATA1, GATA3 and GATA4, or treatment of the GATA6-deficient endoderm with the pro-survival growth factor FGF2. The study by Shi et al. also revealed that GATA6 impairs differentiation of the endoderm and pancreatic cell lineages (Shi et al., 2017).

The loss of endoderm gene expression that we observed in GATA6-depleted cells resembles the phenotype associated with depletion of EOMES during endoderm specification (Arnold et al., 2008; Teo et al., 2011). Depletion of EOMES during the differentiation of huESCs to definitive endoderm resulted in a substantial loss of definitive endoderm mRNAs (Teo et al., 2011). Using ChIP-seq, the authors revealed that many of the genes for which expression in the endoderm was dependent on EOMES also had sequences bound by EOMES, which implied that such genes were directly regulated. Moreover, several of the EOMES-occupied regions were also found to be closely associated with SMAD2/3 binding sites. The authors proposed a model whereby EOMES and SMAD2/3, a downstream effector of Nodal/TGFβ signaling, collaborate to promote expression of endoderm genes and repress mesoderm genes (Teo et al., 2011). This role for EOMES in the mesendoderm is consistent with its expression preceding that of GATA6. However, we also noted that EOMES expression is modestly reduced in the GATA6-depleted endoderm. Whether the similarity of the phenotypes caused by GATA6 or EOMES depletion is due to cooperativity between the two transcription factors or is specifically due to reduction in EOMES expression in GATA6-depleted endoderm remains an open question. A direct comparison of the datasets generated in the GATA6-depleted cells (current study) compared to EOMES-depleted cells (Teo et al., 2011) is difficult because of differences in experimental design between the two studies. However, of the 5226 genes in the genome harboring GATA6-occupied sequences, 64.8% (3389 genes) were also occupied by EOMES (Fig. S5A). Of greater relevance, however, we found that of the 239 genes with enriched endoderm expression that contained GATA6 bound sites, 214 (90%) were also bound by EOMES (Fig. S5B). This extensive co-occupancy of endoderm-expressed genes by these transcription factors may suggest that GATA6 and EOMES work cooperatively to drive endoderm identity. Teo et al. also reported expression data for 295 definitive endoderm genes (Teo et al., 2011) (those investigated in this study), and our analysis revealed that 155 (52%) were dependent on EOMES for expression. Examination of this same set of 295 genes revealed that 159 (54%) were dependent on GATA6. Of the 225 endoderm expressed genes that required either GATA6 or EOMES, we found that expression of 89 (30%) were affected by depletion of either transcription factor (Fig. S5C). These analyses comparing transcriptional profiles imply that GATA6 and EOMES may therefore have both cooperative and independent roles in regulating endodermal gene expression.

Previous studies have demonstrated that the GATA proteins work in conjunction with FOXA transcription factors as ‘pioneer’ factors to establish a genomic environment that defines regions of chromatin poised for expression during hepatic development (Bossard and Zaret, 1998; Cirillo and Zaret, 1999; Iwafuchi-Doi and Zaret, 2016). Although GATA6 has not been studied in this context, GATA4 is capable of binding to highly compacted chromatin, which is consistent with a pioneer factor role for the GATA factors (Cirillo et al., 2002). Pioneer factors are thought to bind to regulatory elements within low signal chromatin without necessarily driving gene expression. By modifying chromatin and repositioning nucleosomes, they can control the competency of a gene to be expressed after the cell receives appropriate signaling (Iwafuchi-Doi and Zaret, 2016). Our data show that GATA6 exhibits broad occupancy throughout the genome including many sites outside of the proximal promoters of genes, which implies that GATA6 commonly binds distal cis-regulatory elements (CREs) or intronic enhancers. Also, many of the genes with sequences bound by GATA6 are not expressed in the endoderm based on our analyses of the transcriptome data (Fig. 4G). The occupancy of silent genes would be consistent with a pioneer factor role for GATA6. Efforts to monitor chromatin remodeling and histone modifications at GATA6 genes in control and GATA6-depleted cells during their differentiation are ongoing and are likely to be informative.

In summary, both GATA4 and GATA6 expression begins 48 h after induction of endoderm from pluripotent stem cells. Surprisingly, depletion of GATA4 was found to have minimal impact on the formation of the endoderm in this cell culture model. However, in contrast to GATA4, GATA6 depletion during endoderm differentiation causes apoptosis of the nascent endoderm beginning 48 h after induction of endoderm differentiation. The apoptosis of the nascent endoderm in GATA6-depleted cells is concomitant with a loss of definitive endoderm gene expression. This broad impact on the expression of endoderm markers is consistent with GATA6 occupancy at a large number of endoderm genes. Several of the GATA6-occupied genes whose expression is reduced following GATA6 depletion encode transcription factors with known roles in controlling endoderm differentiation. Along with EOMES, GATA6 therefore appears to be a central regulator of endoderm fate. The future challenges that arise from this study include developing a better understanding of the mechanism through which GATA6 controls endoderm fate and whether its role as a pioneer factor is central to endoderm formation.

## MATERIALS AND METHODS

### Cell culture and differentiation

H1 (WA01) huESCs (Thomson et al., 1998) were obtained from the WiCell Research Institute, Madison, USA, and cultured under standard conditions on mouse embryonic fibroblasts (MEF) or an E-Cadherin substrate (Nagaoka et al., 2010) in MEF-conditioned medium or mTeSR. Endoderm differentiations were performed as previously described (Mallanna and Duncan, 2013; Nagaoka et al., 2010). The use of human stem cells was approved by the Medical College of Wisconsin Stem Cell Research Oversight committee. H1 huESCs were used to generate the cell lines necessary for experimentation as described previously (Delaforest et al., 2011). For knockdown experiments, GATA4 or GATA6 were depleted from H1 cells using lentiviruses that express shRNAs targeting GATA4 (5′-TGGACATAATCACTGCGTAATTCAAGAGATTACGCAGTGATTATGTCC-3′) or GATA6 (5′-GCGCTGACAGAACGTGATTCTTTCAAGAGAAGAATCACGTTCTGTCAGCGC-3′) (Kanematsu et al., 2007). Calcium phosphate was used to transfect packaging plasmids and either empty plasmid (pLL3.7) or plasmid containing shRNA into HEK293T cells. Virus was collected in MEF-conditioned medium, and huES cells were transduced with the viral-conditioned medium. Polyclonal lines were established by adding selection to the medium 2 days after viral transduction. For rescue experiments, GATA6 cDNA (short isoform) or GATA4 cDNA were cloned into a doxycycline-inducible expression vector. Because the shRNA used in this study targets the 3′ UTR of GATA6, the mouse GATA4 and human GATA6 cDNAs used for the rescue experiments are not sensitive to the GATA6 shRNA. These cDNA plasmids were linearized and electroporated into GATA6-depleted H1 cells and polyclonal lines were selected. For ChIP-seq, 3′ AviTag-tagged GATA6 cDNA and FLAG-tagged BirA (Wang et al., 2009; Kim et al., 2009) were cloned, in frame with each other, separated by a viral T2A sequence (GAGGGCAGAGGAAGTCTTCTAACATGCGGTGACGTGGAGGAGAATCCCGGCCCT), into a doxycycline-inducible vector. A control huESC line had FLAG-tagged BirA cloned into the doxycycline-inducible vector alone. In both rescue and ChIP-seq experiments doxycycline levels were titrated to obtain a one- to fivefold overexpression of GATA6 compared to wild-type cells. In ChIP-seq experiments, control cells were treated with doxycycline at a level such that the BirA expression matched that of the experimental cell line.

### RT-qPCR

RNA was harvested from cells using a Qiagen RNeasy Mini Kit (74104, Qiagen). Total RNA was DNase treated and converted to cDNA using MMLV Reverse Transcriptase (28025-013, Thermo Fisher Scientific). cDNA was used as a template in RT-qPCR reactions with PCR primer and probe sets (Integrated DNA Technologies, Coralville, USA) (Table S1). Reactions were run on an Applied Biosystems StepOne Plus Real-Time PCR machine (4376600, Thermo Fisher Scientific), and data analyses were performed using the SABiosciences RT2 Profiler PCR Array Data Analysis program (http://pcrdataanalysis.sabiosciences.com/pcr/arrayanalysis.php). Statistical significance was determined using Student's *t*-tests (unpaired, two tailed).

### Western blotting

Protein from whole cell lysates was run on 4-12% Tris-Bis acrylamide gels (NP0321, Thermo Fisher Scientific) using the NuPAGE system (Invitrogen). Protein was transferred to PVDF blotting membranes (1620177, Bio-Rad) using wet electroblot apparatus. Blots were blocked with 5% non-fat milk in TBST. Primary and secondary antibodies (Table S2) were applied to the blots in 5% non-fat milk in TBST. Three 5 min TBST washes were performed after each antibody incubation. Following the last wash, SuperSignal West Pico Chemiluminescent substrate (34080, Thermo Fisher Scientific) was applied to the blots for 3 min. The blots were then exposed to films (F-9023, GeneMate, VWR, Radnor, PA), which were developed in a SRX-101A developer (Konica Minolta, Ramsey, NJ). After probing with anti-GATA4 antibodies, blots were stripped using One Minute Western Blot Stripping Buffer (GM Bioscience, Rockville MD) and subsequently re-probed with anti-GATA6 and anti-TBP antibodies (Fig. 1F).

### Immunostaining

Cells were fixed with 4% paraformaldehyde in PBS. Cells were made permeable using 0.5% Triton-X-100 and blocked with 3% BSA in PBS, before primary and secondary antibodies (Table S2) were applied in 1% BSA in PBS, and cells were counterstained with DAPI. Micrographs were taken using an Eclipse TE300 fluorescent microscope (Nikon, Tokyo, Japan) and SpotCamera software. Images were assembled into figures using Adobe Illustrator, and images from control and experimental samples were processed identically.

### Oligonucleotide arrays

RNA (250 ng) was converted to aRNA using a 3′ IVT Express Kit (901228, Affymetrix, Thermo Fisher Scientific, Waltham, MA). Following fragmentation, the aRNA was hybridized to Human Primeview Arrays (901837, Affymetrix) and the chips were washed using a GeneChip Fluidics Station 450 (00-0079, Affymetrix). CEL files were normalized with RMA and ANOVA comparisons were performed using Partek Genomics Suite, Partek Incorporated, St. Louis, Missouri. All arrays used in this study are summarized in Table S3A. Microsoft Excel was used to calculate z-scores and to generate gene lists. The lists included genes that exhibited a z-score cutoff >3 or <−3 and *P*<0.01; z-score summaries for each comparison are shown in Table S3B. Gene lists were uploaded to PANTHER (http://pantherdb.org) and the Broad Institute's Molecular Signatures Database (MSig; http://software.broadinstitute.org/gsea/msigdb/) to identify gene families, molecular pathways and biological processes that were affected by GATA6 depletion during endoderm formation. Heat maps were generated and hierarchical clustering was performed in Partek. Original data have been deposited in the Gene Expression Omnibus Databases: GSE77360, GSE81898, and GSE81901.

### Apoptosis assay

Cell lines were differentiated into endoderm and at each day during differentiation (pluripotent cells=day 0) cells were liberated with Accutase (Sigma-Aldrich), washed with PBS, and suspended in PBS. Cell viability and apoptosis levels were determined using Guava ViaCount Reagent (4000-0040, EMD Millipore) according to the manufacturer’s recommended protocol. Dilutions of the samples were made to ensure the samples were in the linear range of the assay. Populations were defined by nucleation and viability dye exclusion. Statistical significance was determined via Student’s *t*-test (unpaired, two tailed)

### Proliferation assay

Cell lines were differentiated into endoderm and each day during differentiation (pluripotent=day 0) cells were pulsed with EdU (10 μM) for 30 min. The cells were then liberated with accutase, washed with PBS and fixed with 4% PFA in PBS. The cells were then washed 3× with 1% BSA in PBS, and stored at 4°C until all the samples from a round were collected. The cells were then processed for flow cytometry using a Click-iT EdU kit (C10337, Thermo Fisher Scientific) according to the manufacturer’s recommended protocol. Cells were incubated with Click-iT reagent, washed with 1% BSA in PBS, and run through a flow cytometer (Guava easyCyte Flow Cytometer, EMD Millipore, Billerica, MA). Statistical significance was determined via Student's *t*-test (unpaired, two tailed).

### Chromatin immunoprecipitation with high throughput sequencing

BirA-control and GATA6-BirA experimental cell lines were generated and differentiated to day 4 definitive endoderm as described above. Doxycycline was added to the cells for 24 h prior to collection to induce expression of the BirA- and GATA6-AviTag-tagged transgenes. On collection day, the cells were treated as previously described (Wang et al., 2009; Kim et al., 2009). The chromatin was crosslinked by addition of 1% formaldehyde and incubation at room temperature for 10 min. The formaldehyde was quenched by addition of glycine to 125 mM and the cells were washed three times with ice-cold PBS+protease inhibitors. The cells were collected in PBS+protease inhibitors and suspended at 5,000,000 cells per aliquot. Chromatin was sheared to 100–600 bp using a Bioruptor Pico (Diagenode, Denville, NJ), and 10% of the crosslinked sheared cell lysates was saved for input DNA collection. Chromatin bound by GATA6 was precipitated by overnight incubation with Dynabeads MyOne Streptavidin T1 (65062, Invitrogen) at 4°C on a nutator. The following day the tubes were placed on a magnet and the supernatant was removed. The beads were then washed two times with wash buffer I (2% SDS), once with wash buffer II (0.1% deoxycholate, 1% Triton X-100, 1 mM EDTA, 10 mM Tris-HCL), once with wash buffer III (250 mM LiCl, 0.5% NP40, 0.5% deoxycholate, 1 mM EDTA, 10 mM Tris-HCl), and two times with TE buffer (10 mM Tris-HCl, 1 mM EDTA). The DNA was eluted from the beads and the crosslinks were reversed by incubation in SDS elution buffer (1% SDS, 10 mM EDTA, 50 mM Tris-HCl) in a 65°C water bath overnight. The next day the samples were placed on a magnet and the supernatants were collected. The DNA was then purified by phenol-chloroform extraction followed by isopropyl precipitation. DNA was then run on a bioanalyzer (High Sensitivity DNA Chips, Agilent, Santa Clara, CA) to confirm concentration and shearing efficiency. Libraries were generated and high throughput sequencing (Illumina Hi-Seq) was performed by Beijing Genomics Institute, Shenzhen, China. Quality control of the sequencing reads was performed using FASTQC (https://www.bioinformatics.babraham.ac.uk/projects/fastqc/), MACS (https://github.com/taoliu/MACS) and HOMER (http://homer.ucsd.edu/homer/) software. Sequencing reads were aligned with the human genome (hg19) using BowTie2 software, and enriched regions were identified using MACS2 software with False Discovery Rate<0.05, m-fold>5.0. Homer software was used to annotate enriched regions to their nearest gene ±10 kb of the transcriptional start site using the standard human annotation file. HOMER software was also used to identify a GATA6 consensus binding sequence, and for this analysis all 66582 GATA6 peaks were entered. To determine the average distance to transcriptional start sites, all 66582 sites were entered into the CisGenome Suite (http://www.softsea.com/download//CisGenome.html). Original data have been deposited in the Gene Expression Omnibus Databases (accession numbers: GSE77360, GSE81898, and GSE81901).
